# Tumor-suppressing multi-enterobacteria and PD-1/PD-L1 immune checkpoint inhibitor combination improves the outcome of hepatocellular carcinoma therapy

**DOI:** 10.3389/fimmu.2025.1598436

**Published:** 2025-06-20

**Authors:** Dongjuan Wu, Yaohua Fan, Mingsheng Zhang, Xiaoguang Wang, Xuesong He, Xinwei Guo, Yangchen Liu, Chenxi Cao, Zhaoqun Deng

**Affiliations:** ^1^ Department of Oncology, The Second Affiliated Hospital of Jiaxing University, Jiaxing, China; ^2^ Department of Oncology, Tongji Hospital, Tongji Medical College of Huazhong University of Science & Technology, Wuhan, Hubei, China; ^3^ Department of Radiotherapy, Taixing People’s Hospital Affiliated to Yangzhou University, Taixing, Jiangsu, China

**Keywords:** tumor-suppressing multi-enterobacteria, hepatocellular carcinoma, intestinal microbiota, tumor immune microenvironment, anti-programmed death-1 monoclonal antibody, anti-programmed death ligand-1 (PD-L1) monoclonal antibody, immunotherapy

## Abstract

**Objective:**

Programmed death 1 (PD-1) and its ligand PD-L1 inhibitors and cytotoxic T lymphocyte-associated antigen-4 (CTLA-4) monoclonal antibodies have been approved for the treatment of advanced hepatocellular carcinoma (HCC), but the response rates of these immunotherapy are not high, and they are easy to be resistant. Studies have shown that the gut microbiota can significantly influence immune responses and the efficacy of immune checkpoint inhibitors (ICIs). The aim of this study is to investigate whether the combination therapy of Tumor-Suppressing Multi-Enterobacteria (TSME) and PD-L1 inhibitor (atezolizumab) can improve the efficacy of immunotherapy-resistant hepatocellular carcinoma.

**Methods:**

Patients with advanced liver cancer resistant to atezolizumab were treated with tumor suppressor TSME combined with atezolizumab, and the efficacy was evaluated. By establishing a tumor-bearing mouse model, the control group, InVivoMAb anti-mouse PD-1 monotherapy group, TSME group, and anti-PD-1 mab +TSME double drug group were set up. To evaluate whether the combination therapy enhances the antitumor effect, the proportion of T cells in the tumor microenvironment (TME) was analyzed by immunohistochemistry.

**Results:**

Patients with clinically immuno-resistant hepatocellular carcinoma who were treated with TSME still had a PFS of about 7 months with continued atezolizumab treatment, and they were still in long-term survival. The *in vivo* model showed that TSME combined with αPD-1 promoted the efficacy of anti-PD-1 antibody immunotherapy by increasing the proportion of CD8^+^ T cells and CD4^+^ T cells in the tumor microenvironment and reducing the proportion of regulatory T cells (Tregs) compared with TSME alone or αPD-1 alone. The relative tumor inhibition rate (TGI) of αPD-1+TSME combination group was as high as 58.78% ± 7.55%. Tumor volume was lower in the αPD-1+TSME group than in the monotherapy group.

**Conclusion:**

Anti-tumor TSME combined with αPD-1 mAb may be a new strategy to improve the sensitivity of immune-resistant patients with advanced hepatocellular carcinoma to anti-PD-1 immunotherapy.

## Introduction

Primary liver cancer, as a common malignant tumor, is the third leading cause of cancer-related death worldwide, and hepatocellular carcinoma accounts for 75-85% of all cancer-related deaths (1). Surgery is the main method for early-stage liver cancer. However, due to the insidious onset of liver cancer, most patients are in the middle and late stages when they are initially diagnosed, and they miss the opportunity of surgical operation. Moreover, HCC has a high recurrence rate after surgery, with a total recurrence rate of about 70% within 5 years, and most patients lose the chance of reoperation after recurrence (2). Therefore, systemic anti-tumor therapy, especially combination therapy based on immune checkpoint inhibitors (ICIs), is highly recommended. It has become the most used and the most important treatment for unresectable liver cancer. The combination therapy of PD-L1 inhibitors with antiangiogenic agents, specifically the atezolizumab plus bevacizumab regimen, has been recommended by major clinical guidelines as first-line treatment for advanced HCC patients (3). Notably, the immunotherapeutic combination of the PD-1 inhibitor Opdivo (nivolumab) and the CTLA-4-targeting antibody Yervoy (ipilimumab) represents the first approved dual immune checkpoint inhibitor regimen for the management of advanced HCC populations (4). ICIs for the treatment of HCC mainly include cytotoxic T lymphocyte-associated antigen (CTLA) monoclonal antibodies. Inhibitors of programmed death 1 (PD-1) and its ligand PD-L1, which kill tumor cells and inhibit their proliferation by reactivating the immune response of T cells to tumors (5–7). However, only 20 to 40% of cancer patients respond to immunotherapy (8). In a study of Asian HCC patients treated with a single PD-1 immunoagent such as nivolumab, pembrolizumab, or camrelizumab, the objective response rate (ORR) was found to be only approximately 15%, suggesting that the complex immunosuppressive microenvironment of HCC leads to ICIs evasion by unknown mechanisms (5, 9, 10). Their use has been hampered by limited response rates and a lack of predictive markers for clinical response (11, 12). Elucidation of the mechanisms underlying the immunosuppressive microenvironment and subsequent remodeling to guide rational combination therapy remains a major challenge for therapeutic intervention in HCC patients (13). There is an urgent need to overcome the intrinsic or adaptive resistance of HCC to immunotherapy.

Gut microbiota has been shown to play a regulatory role in the response to tumor immunotherapy (14). In recent years, several authoritative studies have shown that the number, type and composition of intestinal flora in cancer patients are closely related to the efficacy and survival of these patients treated with PD-1 inhibitors. The possible principle is that intestinal flora regulates the tumor microenvironment through microbial signals, thereby affecting the efficacy of immunotherapy (15–18). In 2015, two Science papers published startling results showing that gut microbiota plays a decisive role in the response of immunotherapy in mouse models (19, 20). At present, mouse models of different cancers have been studied to enhance the efficacy of ICIs after Fecal bacteria transplantation (FMT), including colorectal cancer (21), malignant melanoma (22), and renal cancer (23). However, in the clinical stage, FMT only achieved good results in patients with malignant melanoma: In 2021, FMT was tried in patients with immune-resistant malignant melanoma, and more than one third of patients experienced a re-response, using FMT from an effective population (22). In another 2023 study, patients with melanoma who received fecal microbiota from a healthy person had an approximately 20% improvement in response rate to first-line immunotherapy, with an objective response rate of 65% (24). In addition, in NSCLC patients, novel prebiotics have been tried to enhance the response to anti-PD-1 immunotherapy in NSCLC patients (25). In HCC, studies have found the relationship between the species diversity and abundance of gut microbiota and the clinical response and adverse effects of immunotherapy (26). Based on the above evidence, we can hypothesize that gut microbiota transplantation can also improve the efficacy of liver cancer immunotherapy.

Based on long-term experimental studies, we have identified specific gut bacteria associated with tumor immunity. In melanoma-bearing mice, the combination of oral *Bifidobacterium* (containing *Bifidobacterium breve* and *Bifidobacterium longum*) with PD-L1 inhibitors reduced tumor size by 80%, whereas PD-L1 inhibitors alone achieved only a 40% reduction (18). In melanoma patients, fecal samples from immunotherapy responders showed enrichment of probiotics like Bifidobacterium. Transplantation of these microbiota into mice enhanced the efficacy of immunotherapy (27). For Chinese non-small cell lung cancer patients receiving *Bifidobacterium breve* supplementation during immunotherapy, the objective response rate (ORR) reached 40%, disease control rate (DCR) 90%, and median progression-free survival (PFS) exceeded 500 days - significantly higher than in *Bifidobacterium breve*-negative patients (28). *Bifidobacterium*-derived signals were found to stabilize dendritic cell (DC) activation, thereby improving tumor-specific CD8^+^ T cell effector functions (19). *Lactobacillus reuteri* has also demonstrated immunotherapeutic enhancement. In melanoma mice, *L. reuteri* transplantation combined with PD-L1 inhibitors resulted in an additional 60% tumor reduction. In advanced melanoma patients, serum levels of the *L. reuteri* metabolite indole-3-aldehyde (I3A) significantly correlated with treatment outcomes, with high-I3A patients showing median PFS >50 months versus <10 months in low-I3A groups (29). Another species, *Lactobacillus johnsonii*, was shown to metabolize hypoxanthine and stimulate immunity via adenosine receptors. In colorectal cancer mice, *L. johnsonii* combined with CTLA-4 inhibitors reduced tumor volume by 85% compared to CTLA-4 inhibitors alone (30). Other immunomodulatory Lactobacillus species including *L. casei* and *L. plantarum* have also demonstrated potential for enhancing cancer immunotherapy (31). *Streptococcus thermophilus* exhibited anti-tumor activity through β-galactosidase secretion, significantly suppressing tumorigenesis in animal models. Notably, this effect disappeared when β-galactosidase-related genes were knocked out (32). Additional studies revealed that oral *Lactobacillus rhamnosus* GG enhanced anti-PD-1 efficacy by increasing tumor-infiltrating DCs and T cells (33).

The TSME used in this study incorporates these validated tumor-immunomodulatory gut bacteria and immune potentiators. Its formulation includes *Bifidobacterium longum*, *Lactobacillus rhamnosus*, *Bifidobacterium animalis*, *Bifidobacterium adolescentis*, *Lactobacillus reuteri*, *Lactobacillus casei*, *Streptococcus thermophilus*, *Bifidobacterium bifidum*, and *Lactobacillus acidophilus*. TSME has obtained China Food Standards certification (Food Production License SC10632117100037), ensuring safety for oral administration and clinical application.

This study aims to investigate whether TSME combined with PD-1 inhibitors can enhance cancer immunotherapy efficacy, particularly in immunotherapy-resistant patients.

## Materials and methods

This research complies with all relevant ethical regulations approved by the Institutional Animal Care and Use Committee (IACUC) of Huazhong University of Science and Technology.

This Investigator-initiated Trial (IIT) was performed under the guidance of Medical Ethics Committee of Taixing People’s Hospital (ethical lot. LS2023019). The subject has signed the informed consent form.

### Tumor-suppressing multi-enterobacteria

TSME is a well-crafted blend of nine strains of intestinal probiotics. Its formulation includes *Bifidobacterium longum*, lactobacillus rhamnosus, Bifidobacterium animalis, bifidobacterium adolescentis, Lactobacillus reuteri, Lactobacillus casei, Streptococcus thermophilus, Bifidobacterium bifidum, and Lactobacillus acidophilus. Each strain has been carefully selected for its potential health benefits, working in synergy to offer a comprehensive approach to supporting gut health and impacting tumor progression. TSME is a food-grade probiotic complex, with Food Production License No. SC10632117100037.

### Cell culture

The murine hepatocellular carcinoma H22 cell line was obtained from Meisen CTCC (Zhejiang, China). H22 cells were cultured in RPMI 1640 medium which was supplemented with 10% FBS and 100 U/mL Penicillin-Streptomycin and incubated with a humidified atmosphere containing 5% CO_2_ at 37°C. RPMI 1640 medium, fetal bovine serum (FBS), Penicillin-Streptomycin, were all purchased from Thermo Fisher Scientific Inc (MA, USA).

### Tumor model

70 female Balb/c mice aged 6–7 weeks (18–22 g) were obtained from Beijing Charles River Laboratory Animal Technology Co., Ltd (Beijing, China) and raised in the animal care facility in the SPF grade environment with sterilized food pellets and distilled water under a 12 h light/dark cycle. All animal studies were performed in accordance with the regulations approved by the Institutional Animal Care and Use Committee (IACUC) of Huazhong University of Science and Technology.

H22 cells (1 × 10^6^) were implanted subcutaneously into the right flank of Babl/c mice. Body weight, maximum length of major axis (L), and maximum length of minor axis (W) of tumors were measured every three days during the whole animal experiment. When the mean tumor volume is approximately 100 mm^3^ after tumor implantation, mice were randomized into four groups (n = 10 in each group) as day 0. Experimental mice were allocated to study groups through computer-generated randomization using the RAND() function in Microsoft Excel (version 16.78). This simple randomization method produced unique random numbers for each subject, followed by rank-order stratification to achieve balanced group allocation. The procedure ensured: 1) Equal sample size across groups (n=10/group); 2) Baseline characteristic homogeneity (weight variance <5%); 3) Allocation concealment through blinded assignment. The four treatment groups were given separately with vehicle, anti PD-1 antibody (InVivoMAb anti-mouse PD-1(CD279)), TSME and anti PD-1 antibody + TSME combination.

Anti PD-1 antibody (5 mg/kg) was intraperitoneally injected once every 3 days. Mice were orally treated with 0.2 mL TSME everyday(10 billion CFU/mL). Tumor growth inhibition (TGI% =(1-T/C)× 100%) of each mouse was calculated. Mice were euthanized when tumor volume reached 2000 mm^3^.

### Immunohistochemistry

The tumor specimens of four groups were fixed in a 10% formalin solution and embedded in paraffin for sectioning at a thickness of 4 μm. Immunohistochemical reactions were carried out with streptavidin-biotin-peroxidase. Sections were deparaffinized in xylene, washed in phosphate-buffered saline (PBS, pH 7.4), and rehydrated through a graded ethanol series. Endogenous peroxidase activity was blocked by incubation in 3% hydrogen peroxide/methanol for 10 minutes, after which the specimens were washed with PBS. Specimens were placed in 10% normal goat serum (Histofine SAB-PO kit, Nichirei Corporation, Tokyo, Japan) for 5 minutes and then incubated at room temperature for 30 minutes with the following primary antibodies: anti-mouse CD4 (Abcam, ab218628, diluted at 1:500), anti-mouse CD8 (Proteintech, 29896-1-AP, diluted at 1:500) and anti-mouse Foxp3 (Biolegend, 126403, diluted at 1:500). After washing in PBS, biotinylated goat anti-rabbit immunoglobulin (Solarbio, Beijing) was applied, and incubated at room temperature for 30 minutes. After washing in PBS, immunohistochemical reactions were developed in freshly prepared 3,3’-diaminobenzidine tetrahydrochloride (Histofine SAB-PO kit, Nichirei). Slides were counterstained with hematoxylin and coverslipped in a systemic mounting medium. Trypsin-EDTA solution, and Phosphate buffered saline (PBS) were all purchased from Thermo Fisher Scientific Inc (MA, USA).

### Evaluation of CD8^+^, CD4^+^ and Treg tumor-infiltrating cells classification

Immunostained sections were evaluated under a microscope (Olympus, Japan). The degree of immune cell infiltration was observed more than 10 independent high-power (×200) microscopic fields for each tissue samples. Then 5 areas with the highest numbers of immune cells were selected in each sample for closer examination. Next, using a microscopic field of ×400, the numbers of immunoreactive cells of each class within cancer cell nests and stroma in these 5 areas were counted; the average of the cell numbers in the 5 fields was used for classification. Image ProPlus software was used for semi-quantitative analysis of Tunel staining results. The average optical density value (IOD/area) = accumulated optical density value/area of the measured staining area, and the larger the value, the stronger the positive. All specimens were evaluated by 2 investigators, If there are differences between the two researchers, they can reach a consensus through discussion or ask the third researcher to make a ruling. The histopathological assessments were conducted under rigorously maintained double-blinded conditions: 1) Sample blinding; 2) Investigator blinding.

### Statistical analysis

Statistical analyses were performed using GraphPad Prism 8.0 software (GraphPad Software Inc). Datas are presented as mean ± standard error of the mean (SEM). Statistical significance for tumor volume between groups was determined by one-way ANOVA. A p-value < 0.05 was considered significant (*p <.05, **p <.01, and ***p <.001).

## Results

### TSME promotes immune checkpoint blockade responsiveness *in vivo*

**Figure 1:**
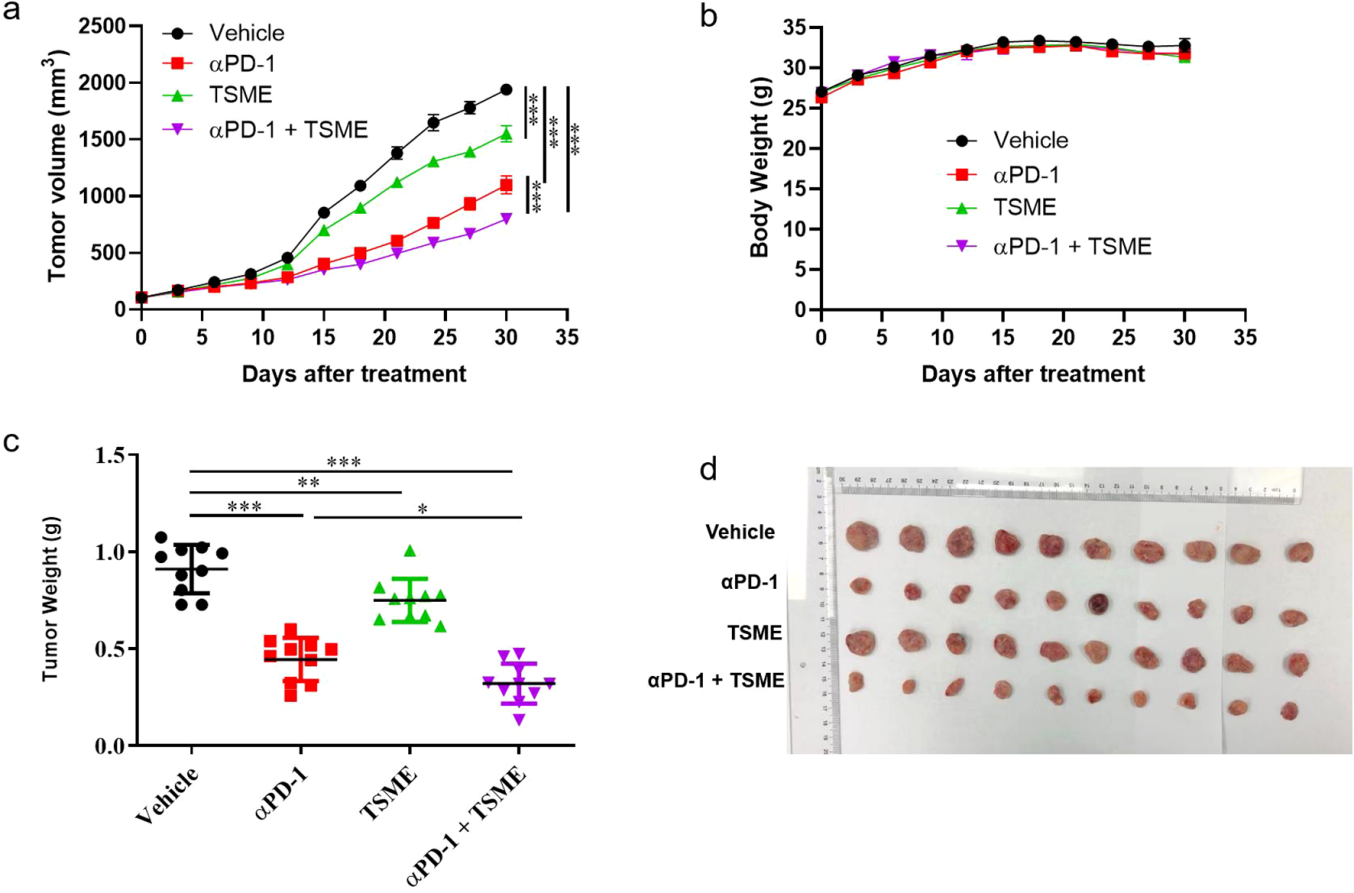
TSME significantly enhanced the anti-tumor effect of αPD-1/αPD-L1 in hepatocellular carcinoma tumor-bearing mice. **(a)** Tumor growth curves of HCC-bearing mice treated with vehicle control, αPD-1, TSME, or a combination of αPD-1 and TSME. Tumor volumes were measured every 3 days post-treatment initiation (n = 10 per group). Data are presented as mean ± SD. Statistical significance was determined by two-way ANOVA (***p < 0.001). **(b)** Body weight monitoring throughout the treatment period showing no significant weight loss across treatment groups, indicating limited systemic toxicity. **(c)** Final tumor weights at the end of the experiment. Combination treatment with αPD-1 and TSME resulted in the greatest reduction in tumor burden. Data are presented as mean ± SD. Statistical significance was assessed by one-way ANOVA (*p < 0.05, **p < 0.01, ***p < 0.001). **(d)** Representative images of excised tumors from each treatment group.

**Figure 2:**
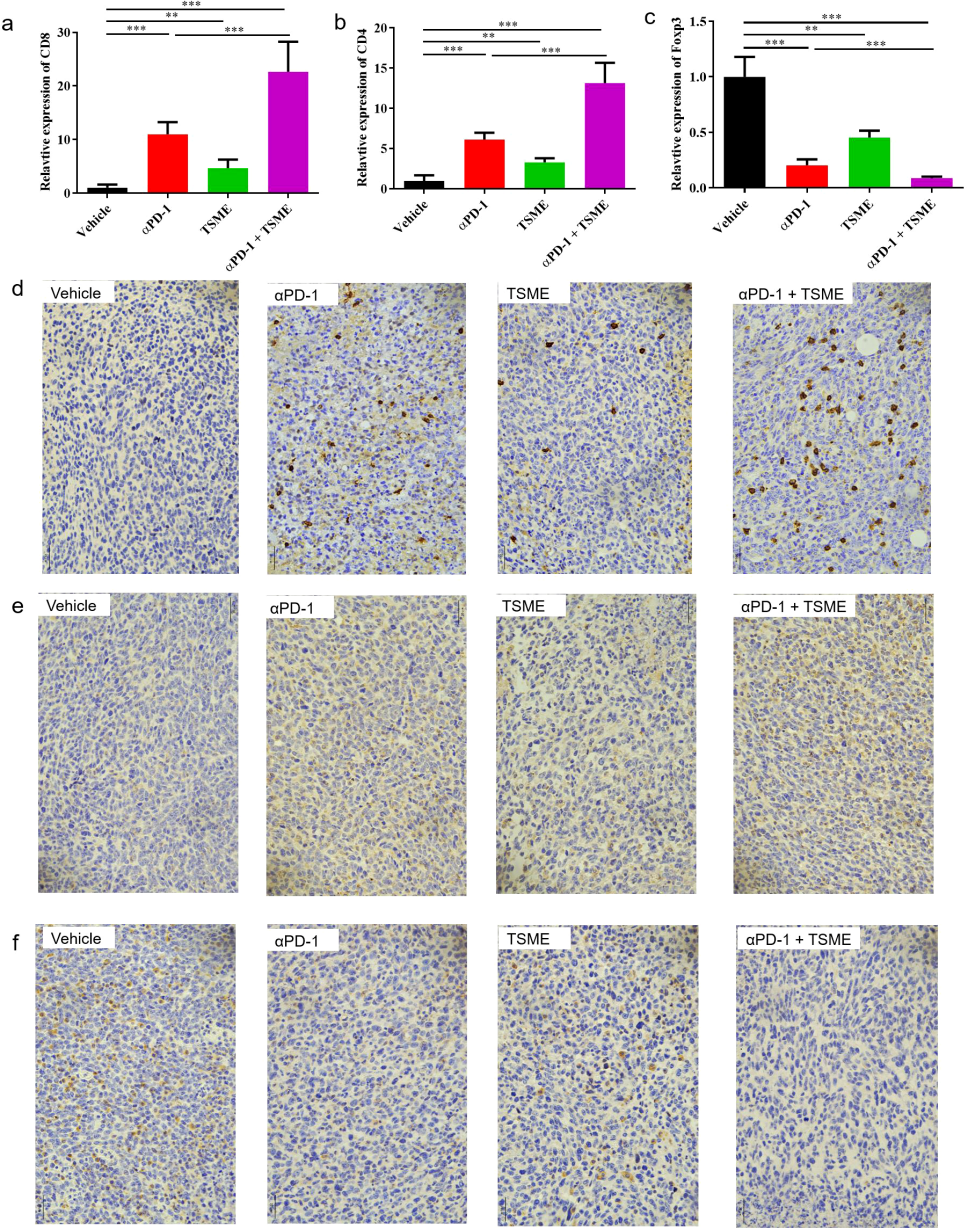
TSME enhances αPD-1-mediated anti-tumor immunity by modulating tumor-infiltrating T cell subsets. **(a–c)** Quantification of tumor-infiltrating CD8^+^ T cells **(a)**, CD4^+^ T cells **(b)**, and Foxp3^+^ Tregs **(c)** in tumor tissues of hepatocellular carcinoma-bearing mice treated with vehicle, αPD-1, TSME, or the combination of αPD-1 and TSME. Data are presented as mean ± SD (n = 10 per group). Statistical analysis was performed using one-way ANOVA followed by Tukey’s *post hoc* test (**p < 0.01, ***p < 0.001). **(d–f)** Representative immunohistochemical (IHC) staining of tumor sections for CD8 **(d)**, CD4 **(e)**, and Foxp3 **(f)** in each treatment group. Brown DAB staining indicates positive immune cell infiltration, while nuclei were counterstained with hematoxylin (blue).

To investigated whether Tumor-Suppressing Multi-Enterobacteria improves the responsiveness of immunotherapy targeting the PD-1/PD-L1 axis in hepatocellular carcinoma, we established the murine hepatocellular carcinoma H22 cell line-derived xenograft subcutaneous tumor. The *in vivo* model demonstrated that TSME significantly enhanced the efficacy of αPD-1 immunotherapy (**Figure 1a**). Compared with vehicle group, the tumor growth inhibition of αPD-1 and TSME groups were 43.30% ± 12.15% and 19.63% ± 12.98%respectively, and the TGI of αPD-1 + TSME group was up to 58.78% ± 7.55%(**Supplementary Figure S3**). As shown in **Figure 1b**, there was no significant difference in mouse body weight among the groups, indicating that both monotherapy and combination therapy had good safety. At the end of the experiment, the mice were euthanized, and the tumors were separated and weighed. The tumor volume of the αPD-1 + TSME group was lower than that of vehicle or any monotherapy group (**Figures 1c, d**). As shown in **Figures 2a, d**, TSME in combination with blockade PD-1/PD-L1 increased the frequency of infiltrating CD8^+^ T cells. As cytotoxic T lymphocytes (CTLs), the more of CD8^+^ T cells (the brown one in **Figure 2d**) often mean more effective against tumors. We also verified the frequency of infiltrating CD4^+^ T cells in the tumors. The proportion of CD4^+^ T cells in the αPD-1 + TSME combination group was higher than that in the any monotherapy group or vehicle group (**Figures 2b, e**), similar to the change of CD8^+^ T cells. In addition, both αPD-1 and TSME decreased the frequency of regulatory T cell in the tumor microenvironment and demonstrated a synergistic effect (**Figures 2c, f**). The αPD-1 + TSME group showed a lower proportion of Treg than any other group.

Collectively, these results demonstrate that modulating the immunosuppressive microenvironment through intestinal flora regulation may be a promising approach to reinforce the effectiveness of tumor ICB therapy in clinical settings.

### Clinical response in a recurrent and metastatic advanced liver cancer patient enrolled on an investigator-initiated trial

**Figure 3:**
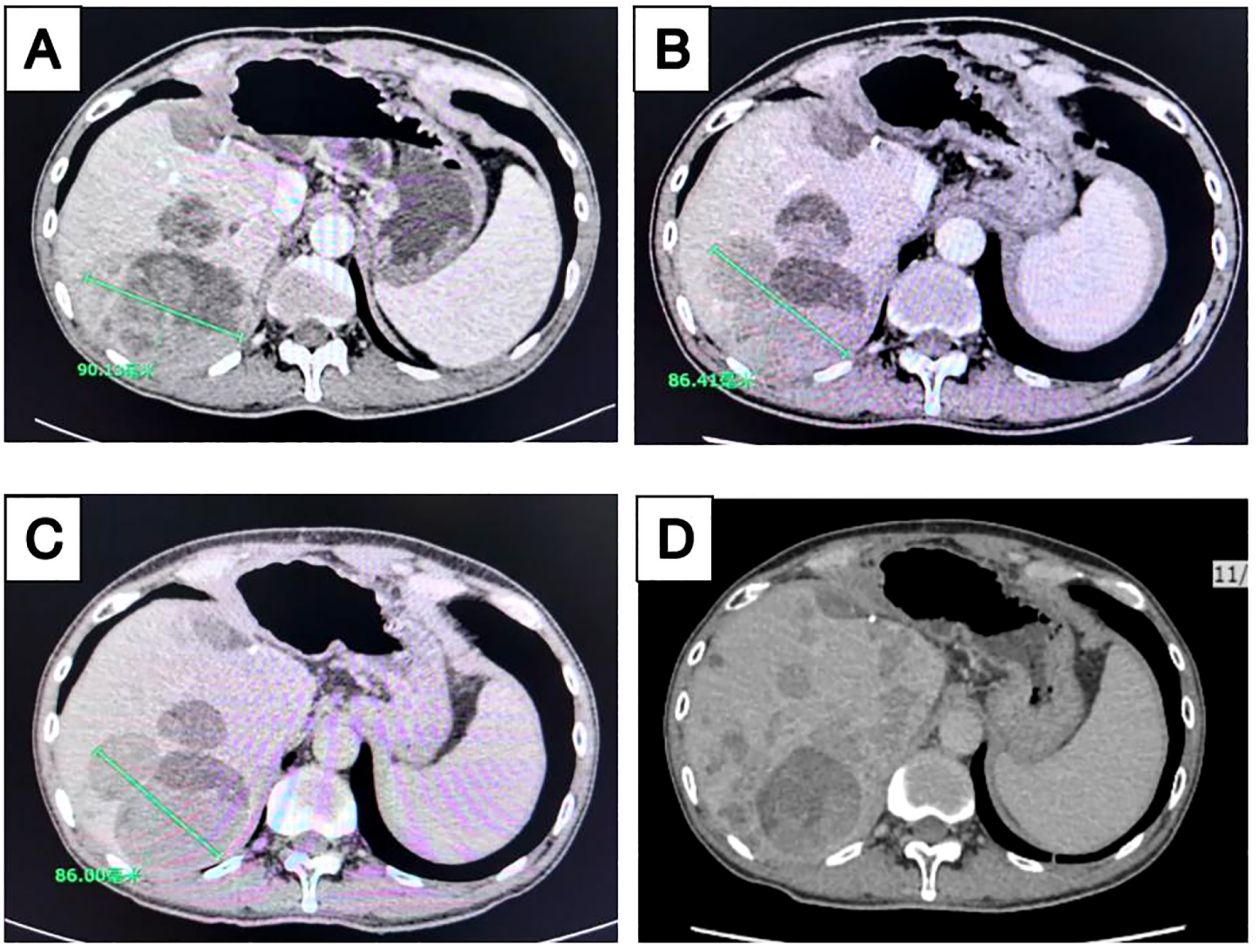
Longitudinal imaging assessment of liver lesions following treatment. Contrast-enhanced computed tomography (CT) scans of the liver acquired at multiple time points during treatment: **(A)** April 2022, **(B)** June 2022, **(C)** August 2022, and **(D)** November 2022.

In December 2019, a patient over 50 years old was diagnosed with hepatocellular carcinoma (**Supplementary Figure S1**). Despite multimodal therapies including transarterial chemoembolization (TACE), left hepatectomy with cholecystectomy, microwave ablation of right hepatic lobe tumors, lenvatinib combined with camrelizumab, hepatic radiotherapy, and lapatinib plus camrelizumab, the disease continued to progress. From December 16, 2021, to January 6, 2022, the patient received two cycles of atezolizumab combined with bevacizumab, yet disease progression persisted (**Figure 3A**). Subsequent maintenance therapy with tegafur-gimeracil-oteracil (S-1) for three months was accompanied by moderate abdominal distension and fatigue.

In April 2022, the patient enrolled in a clinical trial titled “The Efficacy and Safety of Tumor-Suppressing Multi-Enterobacteria Transplantation Combined with Immune Checkpoint Inhibitors in the Treatment of Recurrent or Metastatic Advanced Liver Cancer” (Ethics Approval Number: LS2023019, Medical Ethics Committee of Taixing People’s Hospital). From April 25, 2022, to November 2022, the patient underwent treatment with atezolizumab (1.2 g qd), bevacizumab (15 mg/kg), and TSME (6 capsules bid, days 1-10) every 3 weeks. Radiographic assessments conducted every two cycles demonstrated stable disease with tumor shrinkage (SD-S) (**Figures 3B, C**). Concurrently, the patient exhibited significant alleviation of abdominal distension and fatigue. However, follow-up abdominal CT on November 17, 2022, revealed increased hepatic lesions indicating disease progression (**Figure 3D**), with a progression-free survival (PFS) of 7 months. No significant adverse reactions were observed during TSME administration.

**Figure 4:**
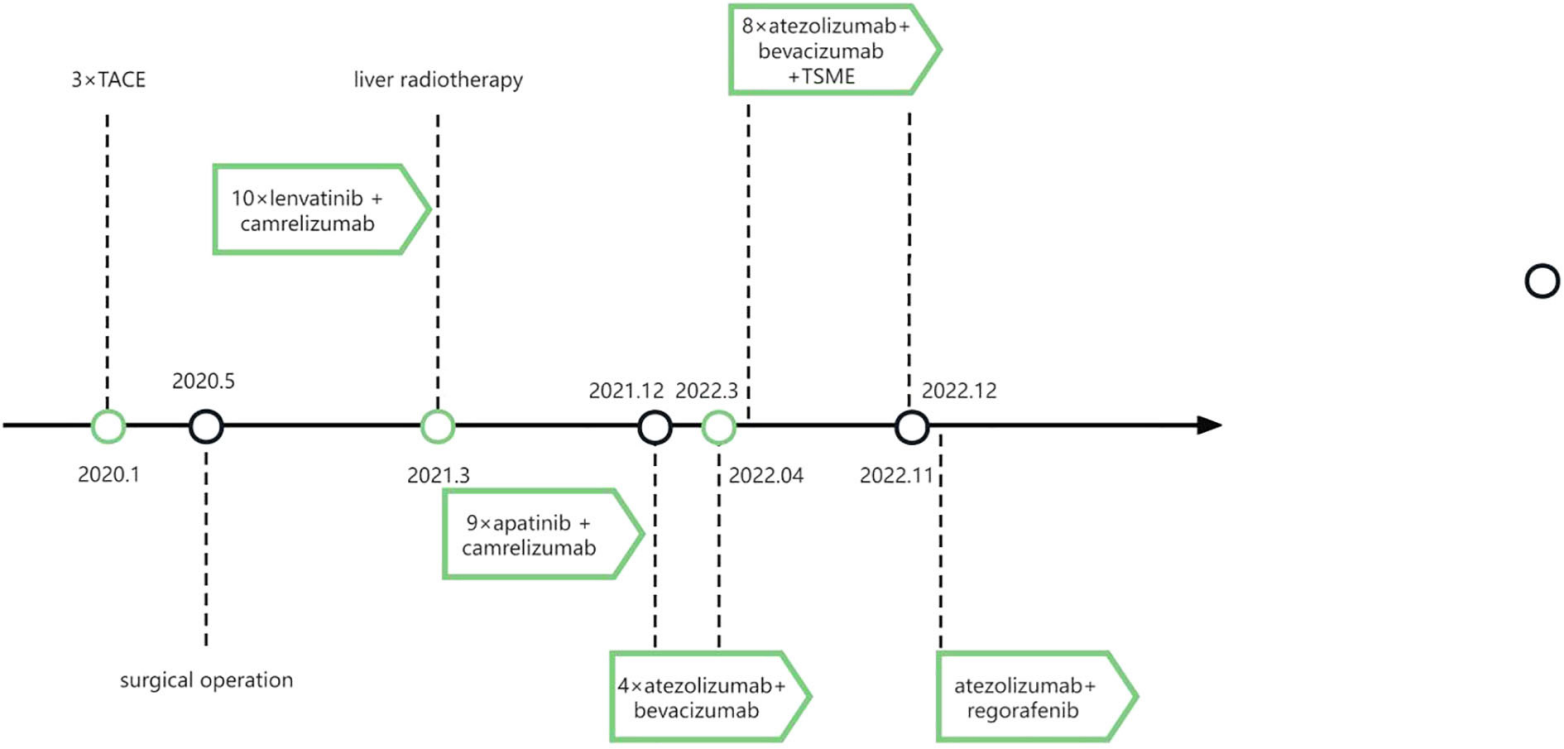
Timeline of the patient’s treatment plans. TACE, trans-arterial chemoembolization. TSME, Tumor-Suppressing Multi-Enterobacteria.

Following disease progression in November 2022, the treatment regimen was switched to lapatinib plus regorafenib. To date, the patient remains in stable condition with preserved quality of life (**Supplementary Figure S2**). The therapeutic timeline is summarized in **Figure 4**.

## Discussion

This study is the first to demonstrate the synergistic antitumor effects of TSME combined with PD-1/PD-L1 inhibitors in immunotherapy-resistant HCC. In animal experiments, TSME significantly enhanced the efficacy of αPD-1 monoclonal antibody by remodeling the tumor immune microenvironment (TME): The combination therapy group exhibited increased infiltration of CD8^+^ and CD4^+^ T cells (**Figures 2a, b**) and reduced proportions of immunosuppressive Treg cells (**Figure 2c**). This immune reprogramming correlated with a tumor growth inhibition (TGI) rate of 58.78%, surpassing monotherapy outcomes (**Supplementary Figure S3**). Clinically, a patient with advanced HCC refractory to multiple therapies achieved a PFS of 7 months and alleviated symptoms (abdominal distension, fatigue) after TSME combined with atezolizumab treatment (**Figures 3**, **4**). Despite eventual disease progression, the patient maintained a stable quality of life, suggesting that TSME may reverse immunotherapy resistance through multispecies synergy.

In this clinical case, the patient received carilizumab therapy for 17 months until disease progression, followed by combination therapy with atezolizumab and bevacizumab. Upon recurrence, the patient was treated with tegafur-gimeracil-oteracil (S-1) for 3 months. Subsequent recurrence prompted combination therapy with oral TSME, atezolizumab, and bevacizumab, which demonstrated favorable clinical efficacy. However, the potential delayed pseudo-progression effect of atezolizumab must be ruled out. Existing studies report varying pseudo-progression rates across tumor types: 2.78%-9.69% in melanoma, 1.81%-5.77% in non-small cell lung cancer, and 2.86%-8.82% in renal cell carcinoma, while HCC has limited documented cases (34). Pseudo-progression typically occurs within the first few weeks of immunotherapy (occasionally up to 12 weeks) but rarely manifests after prolonged treatment. In our case, disease progression was observed 16 weeks after initiating atezolizumab, prompting TSME combination therapy. Thus, the likelihood of atezolizumab-induced delayed pseudo-progression is minimal based on both temporal pattern and incidence rates. Notably, the patient reported moderate abdominal distension and fatigue prior to TSME therapy, which gradually resolved post-TSME intervention. This symptomatic improvement aligns with radiographic stabilization (7-month PFS), strongly suggesting that the observed clinical benefit was primarily driven by the synergistic effects of TSME combined with anti-PD-L1 inhibitors rather than delayed pseudo-progression.

FMT remains a primary strategy for modulating gut microbiota to enhance immunotherapy. However, its clinical utility is limited by donor dependency, batch heterogeneity, and infection risks. For instance, FMT trials in melanoma reported objective response rates (ORR) of only 20–35% (22, 24), with efficacy highly donor-dependent. In contrast, TSME comprises nine well-characterized probiotic strains (e.g., *Bifidobacterium longum*, *Lactobacillus reuteri*), validated in preclinical studies (**Figures 2d-f**). Standardized production (SC10632117100037) ensures batch consistency and food-grade safety for long-term oral administration. Moreover, TSME has a long shelf life of 24 months and is easy to store. The recommended storage conditions are to keep it in a cool, dry place, preferably refrigerated at 4°C, while avoiding direct sunlight. It is also convenient to use: the recommended administration method is to take it with warm water, 6 tablets twice daily. Importantly, the bacterial strains in TSME exhibit acid and bile salt resistance. TSME is prepared using a multi-layer encapsulation technology to prevent degradation by gastric acid and bile, significantly enhancing its suitability for clinical application and widespread promotion. Mechanistically, TSME targets complementary pathways through multispecies synergy: Bifidobacterium activates dendritic cells (DCs) via short-chain fatty acids (SCFAs) to promote CD8^+^ T cells activation (19), while *Lactobacillus reuteri*-derived indole-3-aldehyde (I3A) suppresses Treg differentiation and sustains effector T-cell function (29. Such precision is unattainable with FMT’s heterogeneous microbiota.

The resistance of (HCC) to (ICIs) is closely associated with T cell exhaustion and infiltration of immunosuppressive cells within the tumor microenvironment (TME) (35). Our study demonstrates that the combination of TSME with αPD-1 significantly enhances intra-tumoral CD8^+^/CD4^+^ T cell density (2.1-fold increase compared to monotherapy) while reducing the proportion of Treg cells (**Figures 2c, f**). These findings align with recent evidence suggesting that gut microbiota modulation, such as enrichment of *Lachnospiraceae*, improves HCC prognosis by promoting CD8^+^ T cell infiltration (26). Further supporting this mechanistic link, Mao et al. (26) identified specific bacterial taxa (e.g., *Lachnospiraceae bacterium-GAM79* and *Alistipes sp. Marseille-P5997*) whose abundance correlates with prolonged PFS and overall survival (OS) in immunotherapy recipients (26). Importantly, our work extends these observations by revealing that multi-strain probiotic combinations can systemically remodel the immunosuppressive TME. This immunomodulatory effect may be partially mediated by Alistipes-derived metabolites, which have been shown to inhibit PD-1-induced T cell exhaustion (36).

Clinically, the observed radiological stabilization (SD-S) and symptomatic improvement in TSME-treated patients correlated with dynamic immune reconfiguration in the TME (**Figure 3**). To advance these findings, future studies should employ integrated multi-omics approaches (metagenomics/metabolomics) to establish causal relationships within the “microbiota-metabolite-immune phenotype” axis and refine therapeutic protocols through mechanistic validation.

This study demonstrates that TSME combined with PD-1/PD-L1 inhibitors significantly improves outcomes in immunotherapy-resistant HCC. However, several limitations should be noted. First, clinical evidence relies on a single case, necessitating larger cohorts to validate TSME’s generalizability. Second, the specific contributions of individual TSME strains and their immunomodulatory metabolites require further exploration. Additionally, the H22 cell line-derived subcutaneous xenograft mouse model used in this study exhibits differences in tumor microenvironment characteristics (e.g., immune cell composition, stromal features) compared to human HCC orthotopic tumors, which may limit clinical translatability. Future studies should integrate multi-omics technologies (e.g., metabolomics) to establish causal “microbiota-metabolite-immune phenotype” relationships and explore TSME synergies with other ICIs (e.g., CTLA-4 inhibitors) or targeted therapies.

In conclusion, TSME combined with PD-1/PD-L1 inhibitors offers a novel strategy to overcome immunotherapy resistance in HCC by remodeling the TME. Its standardized formulation and mechanistic precision address FMT limitations, advancing gut microbiota interventions in precision oncology. The rigorous pseudo-progression assessment framework herein provides critical guidance for future clinical trials.

## Data Availability

The original contributions presented in the study are included in the article/**Supplementary Material**. Further inquiries can be directed to the corresponding authors.
