# Supplementary material for: Tumor-suppressing multi-enterobacteria and PD-1/PD-L1 immune checkpoint inhibitor combination improves the outcome of hepatocellular carcinoma therapy
Source: Front Immunol. 2025 Jun 20;16:1598436. doi: 10.3389/fimmu.2025.1598436 (PMC12226584; doi:10.3389/fimmu.2025.1598436)
Supplement: Supplementary file 1 [file DataSheet1.pdf]

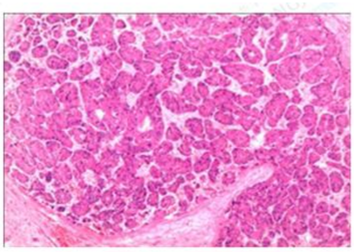

Figure S1. Histopathological evaluation of tumor tissue by hematoxylin and eosin (H&E) staining. (×100)

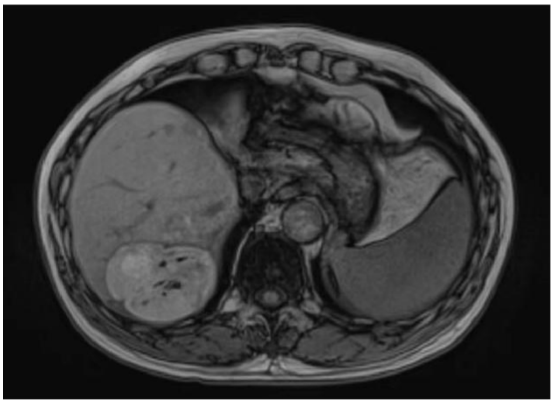

Figure S2 The liver MRI image of the patient in 2024.

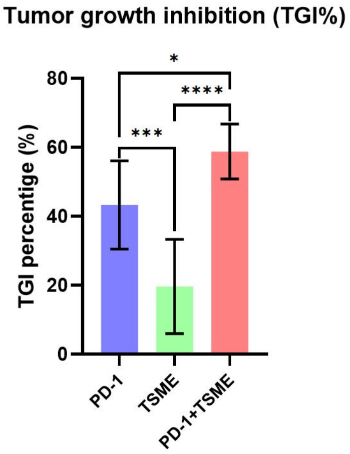

Figure S3 Tumor growth inhibition (TGI%) compared to vehicle control.

Bar graph representing the tumor growth inhibition (TGI%) in different treatment groups, calculated relative to the vehicle control. Mice were treated with PD-1 antibody alone, TSME alone, or a combination of PD-1 and TSME. The combination therapy group exhibited significantly higher TGI% compared to monotherapies, indicating a synergistic anti-tumor effect.

Data are presented as mean ± SD. Statistical significance was assessed by one-way ANOVA with multiple comparisons (*$p < 0.05$, ***$p < 0.001$, ****$p < 0.0001$).
